# Diversity, Daily Activity Patterns, and Pollination Effectiveness of the Insects Visiting *Camellia osmantha*, *C. vietnamensis*, and *C. oleifera* in South China

**DOI:** 10.3390/insects10040098

**Published:** 2019-04-02

**Authors:** Wei Wei, Haipan Wu, Xueyuan Li, Xing Wei, Wen Lu, Xialin Zheng

**Affiliations:** 1 Guangxi Forestry Research Institute, Guangxi Key Laboratory of Special Non-wood Forest Cultivation & Utilization, Improved Variety and Cultivation Engineering Research Center of Oiltea Camellia in Guangxi, Nanning 530002, Guangxi, China; lazioww@163.com; 2 Guangxi Key Laboratory of Agric-Environment and Agric-Products Safety, National Demonstration Center for Experimental Plant Science Education, College of Agriculture, Guangxi University, Nanning 530004, Guangxi, China; haipanwu@163.com (H.W.); m17376042046@163.com (X.L.); 18377174281@163.com (X.W.); luwenlwen@163.com (W.L.)

**Keywords:** visiting insects, hymenoptera, Diptera, cross-pollination, fruit production rate

## Abstract

*Camellia* spp., which are self-incompatible plants, are some of the most important woody species producing edible oil in Southeast Asian countries. However, the demand for camellia oil currently exceeds the supply due to low product yields that have resulted from a decrease in pollination services. Although *Camellia osmantha*, *C. vietnamensis*, and *C. oleifera* are cultivated in South China, little is known about the correspondence between pollinator abundance and pollinator services for this plant genus. In this study, the diversity, daily activity patterns, and pollination effectiveness of insects visiting *C. osmantha*, *C. vietnamensis* and *C. oleifera* were investigated. A total of 24 species, belonging to four orders and 11 families, of visiting insects were identified. *Apis cerana cerana* Fabricius, *Vespa bicolor* Fabricius, *V. velutina* Lepeletier, *V. ducalis* Smith, and *Phytomia zonata* Fabricius were the dominant pollinators. The daily activity peaks of the five visiting insects were between 10:00 and 14:00, which may have been related to the pattern of floral resource production (particularly nectar). Cross-pollination by insects significantly increased the fruit production rates of *C. osmantha*, *C. vietnamensis*, and *C. oleifera*. Therefore, the wild bees and flies that pollinate wild and cultivated *Camellia* plants should be protected in South China.

## 1. Introduction

The fruit and seed set of many crops rely on pollination through a pollinator, especially by insects. Approximately 35% of global crop production arises from crop species that benefit from insect pollination [1]. As is well known, honey bees are the most ubiquitous and commonly used managed pollinator in the world. However, apart from a few managed bee taxa, the great majority of other pollinators (e.g., flies, moths, butterflies, and beetles) that are free-living or wild also provide an ecosystem service to crops [2,3]. Pollination involves interactions between two predominant groups of organisms: the flowering plants and the vectors of their gametes. Such interactions broadly comprise one of the most varied and widespread of all mutualistic relationships [4]. Generally, plants exhibit a remarkable diversity of floral traits that evolve in response to natural selection by a pollinator [5,6]. Therefore, a large number of studies have examined the effects of floral traits, including floral color, scent and nectar, flower number and size, corolla size, etc., on pollinator attraction [7]. For example, evidence was found that increases flower number and size cause increased visitation by syrphid flies [6,7]. Understanding the abundance and identity of insect flower visitors and floral traits on pollinator attraction is an important component in quantifying pollination within crops dependent on insect pollination [8].

*Camellia* spp., which are native to China, are some of the most economically important trees in Southeast Asian countries, as their seeds are used to produce edible tea oil [9]. Indeed, seeds of *Camellia* spp. have been utilized in China for more than 2000 years [10]. The unsaturated fatty acids content of *Camellia* spp. seeds exceeds 84%; therefore, the oil produced from these seeds has been recommended as a healthy edible oil by the Food and Agriculture Organization [11]. However, the demand for camellia oil currently exceeds the supply due to the low product yield that has resulted from a decrease in pollination services [12,13], and *Camellia* spp. reproduction is prevented by a shortage of effective pollinators in some regions, e.g., the plateau region of China [14]. Therefore, it is necessary to investigate pollinator abundance and the corresponding pollination services provided to *Camellia* to identify targets for conservation.

*Camellia osmantha* Ye, Ma et Ye was first recorded in the city of Nanning in Guangxi and described in 2012 as a new species. *Camellia osmantha* is a small evergreen woody tree approximately 4–8 m in height. The trees bloom from October to December [15]. *Camellia vietnamensis* Huang is widely cultivated in tropical and southern subtropical regions including Guangxi, Guangdong, and Hainan in China, as well as Vietnam and Thailand. *Camellia vietnamensis* blooms from November to January [16]. *Camellia oleifera* Abel is widely cultivated in South China as a commercial crop, and its seeds contain abundant edible oil [17]. *Camellia oleifera* blooms from October to January [18]. *Camellia* spp. are insect-pollinated plants, and their recorded insect visitors span five orders, 33 families, and 130 species [13]. For example, in Guangxi Province, 19 families and 54 species of insect visitors of *C. oleifera* have been recorded [19,20]; in Hunan Province, six families and six species have been recorded [21]; and in Fujian Province, 11 families and 24 species have been recorded [22]. The fruit production rates of *C. oleifera* increase significantly after cross-pollination by insects. For example, the fruit production rates increased 2 to 4.5 times after cross-pollination by *Colletes gigas* Cockerell [23] and *Apis cerana cerana* Fabricius [24]. However, little is known about the correspondence between pollinator abundance and pollinator services for *C. osmantha* and *C. vietnamensis*. Such information will be helpful for improving *Camellia* spp. yield and protecting their pollinating insects.

The objective of this study was to investigate the diversity, daily activity patterns and pollination effectiveness of the insects visiting *C. osmantha*, *C. vietnamensis*, and *C. oleifera*.

## 2. Materials and Methods

### 2.1. Study Site

This study was carried out at the Improved Variety and Cultivation Engineering Research Center of Oiltea Camellia of the Guangxi Forestry Research Institute (22°13′–23°32′ N, 107°45′–108°51′ E, 230 m above sea level). *Camellia osmantha* (16,000.0 m^2^), *C. vietnamensis* (13,000.0 m^2^), and *C. oleifera* (20,000.0 m^2^) fields were selected. The trees were 4–6 years old, with heights ranging from 1.8–2.5 m and an average crown size of 2.1 m × 2.0 m. Pesticides were not applied.

### 2.2. The Diversity and Daily Activity of Insects Visiting C. osmantha, C. vietnamensis, and C. oleifera

Insects visiting *C. osmantha*, *C. vietnamensis*, and *C. oleifera* were observed between 08:00 and 18:00 during the period of flowering. Within each *Camellia* ssp. field, 20 trees were randomly observed for ≈ 30 min (excluding trees at field edges) on 23–24 November 2016, 10–11 January 2017, and 22 December 2017. Data were pooled across the two flowering seasons, resulting in a total of ~30 observation hours for 60 trees of *C. osmantha*, *C. vietnamensis*, and *C. oleifera*. All visiting insects were first photographed with a professional single-lens reflex (SLR) camera (EOS 5D Mark II, Canon, Tokyo, Japan). Some species that were not identified in loco were collected using an insect net, and identification of the collected insects was performed using keys in the laboratory [25,26,27]. The total number of insects visiting *C. osmantha*, *C. vietnamensis*, and *C. oleifera* was recorded.

Within each *Camellia* ssp. field, four trees were randomly observed between 08:00 and 18:00 on 25–26 December 2017 and 10–11 January 2018. Floral visitors, including the number of flowers visited, were recorded hourly and pooled across the two sampling dates, resulting in a total of ~120 observation hours for 12 trees of *C. osmantha*, *C. vietnamensis*, and *C. oleifera*.

### 2.3. Floral Traits of C. osmantha, C. vietnamensis, and C. oleifera

**Figure 1:**
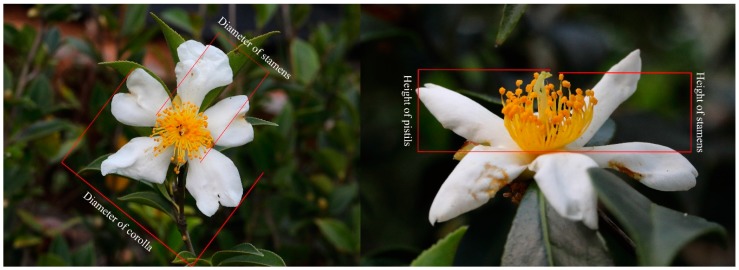
The measurement position for the diameter of the corolla and stamens and the height of stamens and pistils of *Camellia* ssp. flowers.

To explore the potential effect of floral traits on attraction to pollinator visitation, a total of 30 flowers from 10 trees (three flowers/tree) within each *Camellia* ssp. field were randomly selected to measure the diameter of the corolla, height, and diameter of the stamens, and height of the pistils with a Vernier caliper at their full-bloom stage (Figure 1).

### 2.4. Nectar Volumes and Sugar Concentrations of C. osmantha, C. vietnamensis, and C. oleifera Flowers

Nectar availability and sugar concentration were determined by sampling flowers at midday from plants within the area of pollinator counts. The nectar volume present in one flower was always too low to determine volume and sugar concentration on a per flower basis. Therefore, volume and concentration measurements were taken on pooled nectar samples from a number of flowers [28]. A total of 5–8 flowers per tree were collected for measurement to determine the dynamic changes of nectar volume and sugar concentration from the 1st (full-bloom stage) to the 5th day (end of blooming). Nectar volume was measured a calibrated micropipette (Rainin, New York, NY, USA) and concentration was measured with a hand saccharimeter (Atago, Tokyo, Japan). Three replications and a total of 111, 57, and 117 flowers were used for *C. osmantha*, *C. vietnamensis*, and *C. oleifera* measurements, respectively.

### 2.5. Effect of Insect Pollination on the Fruit Production Rates of C. osmantha, C. vietnamensis, and C. oleifera

To determine the pollination effectiveness of the insects, five trees of *C. osmantha*, *C. vietnamensis*, and *C. oleifera*, respectively, were selected randomly in the fields. On each of the 15 trees, one branch was enclosed in a white nylon net cage (length× width × height = 100 × 100 × 100 cm, mesh: 0.1 mm) before the onset of flowering to prevent pollination by insects (treatment group). Another branch of similar size at each tree was marked and left uncaged to allow pollination by insects (control group). The number of buds was recorded at all branches to calculate fruit production rates (number of fruits/number of buds × 100%) per branch. The cages were removed after fruit had developed.

### 2.6. Statistical Analyses

Statistical analyses were performed using SPSS 16.0 (SPSS Inc., Chicago, IL, USA). Species richness and total flower visitor abundance as well as abundance of dominant flower visitors were compared among *C. osmantha*, *C. vietnamensis*, and *C. oleifera* flowers by one-way analysis of variance (ANOVA), followed by Tukey’s honest significant difference (HSD) test for multiple comparisons. The tendency of dominant flower visitors towards flower constancy was evaluated by comparing the percentage of visits of each dominant visiting insect to 1, 2, and ≥3 flower(s) on one *Camellia* tree by one-way ANOVA, followed by Tukey’s HSD test for multiple comparisons. The diameter of the corolla, height and diameter of the stamens, height of the pistils, nectar volume, and sugar concentration were compared among *C. osmantha*, *C. vietnamensis*, and *C. oleifera* flowers by one-way ANOVA, followed by Tukey’s HSD test for multiple comparisons. The fruit production rates were compared between the treatment group and the control group for *C. osmantha*, *C. vietnamensis*, and *C. oleifera* separately using a paired samples *t*-test. Proportional data were subjected to arcsine square root transformation prior to analysis. A level of *p* < 0.05 was accepted as statistically significant for all statistical analyses.

## 3. Results

### 3.1. The Diversity and Daily Activity of Insects Visiting C. osmantha, C. vietnamensis, and C. oleifera

**Figure 2:**
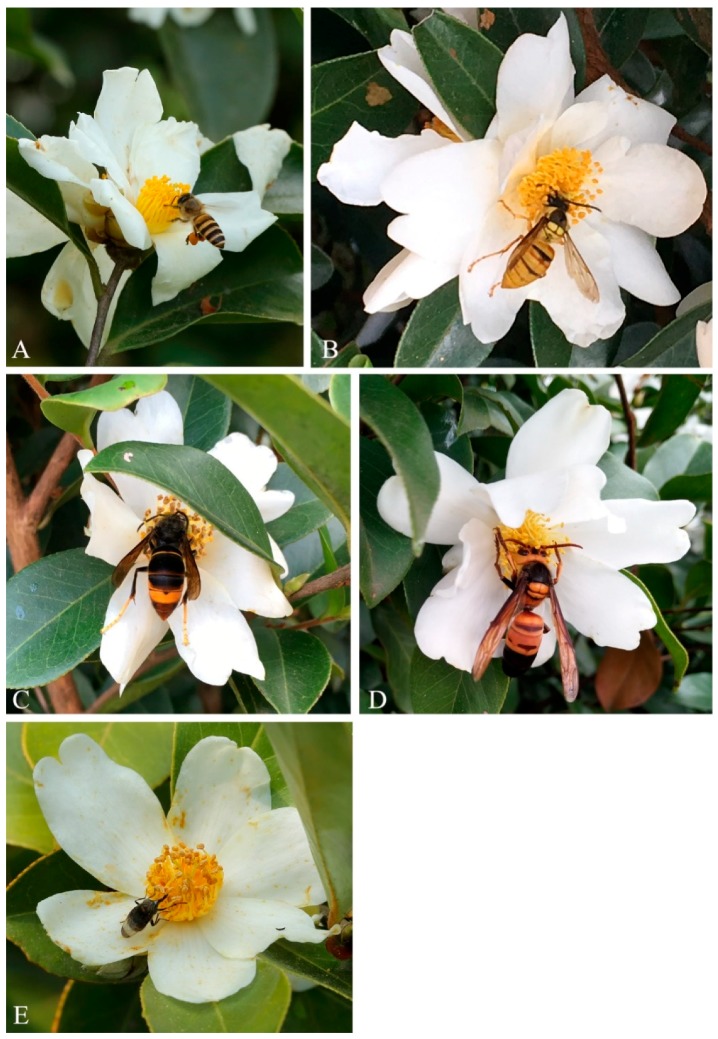
Insects visiting *Camellia* flowers. (**A**) *Apis cerana cerana*; (**B**) *Vespa bicolor*; (**C**) *Vespa velutina*; (**D**) *Vespa ducalis*; (**E**) *Phytomia zonata*.

A total of 24 species, belonging to four orders and 11 families, of visiting insects were identified (Table 1). The dominant species of visiting insects were *A. cerana cerana* Fabricius, *Vespa bicolor* Fabricius, *V. velutina* Lepeletier, *V. ducalis* Smith, and *Phytomia zonata* Fabricius (Figure 2).

There was no significant difference in the number of flower visits (visits/day) for *A. cerana cerana* (F = 1.032, df = 2,11, *p* = 0.395), *V. velutina* (F = 0.454, df = 2,11, *p* = 0.649), or *P. zonata* (F = 1.491, df = 2,11, *p* = 0.276) on *C. osmantha*, *C. vietnamensis*, and *C. oleifera*. However, the number of visits by *V. bicolor* (F = 4.66, df = 2,11, *p* = 0.041) and *V. ducalis* (F = 7.071, df = 2,11, *p* = 0.014) to *C. oleifera* was significantly higher than that to *C. osmantha* and *C. vietnamensis* (Table 2).

The percentage of visiting insects that consecutively visited ≥3 flowers on one tree was significantly higher than the percentage that visited one flower or two flowers for *A. cerana cerana* (F = 41.381, df = 2,8, *p* = 0.0003) but not *V. bicolor* (F = 10.741, df = 2,8, *p* = 0.01) and *V. velutina* (F = 7.203, df = 2,8, *p* = 0.025), *V. ducalis* (F = 34.696, df = 2,8, *p* = 0.0005), or *P. zonata* (F = 4.191, df = 2,8, *p* = 0.073) (Table 3).

**Figure 3:**
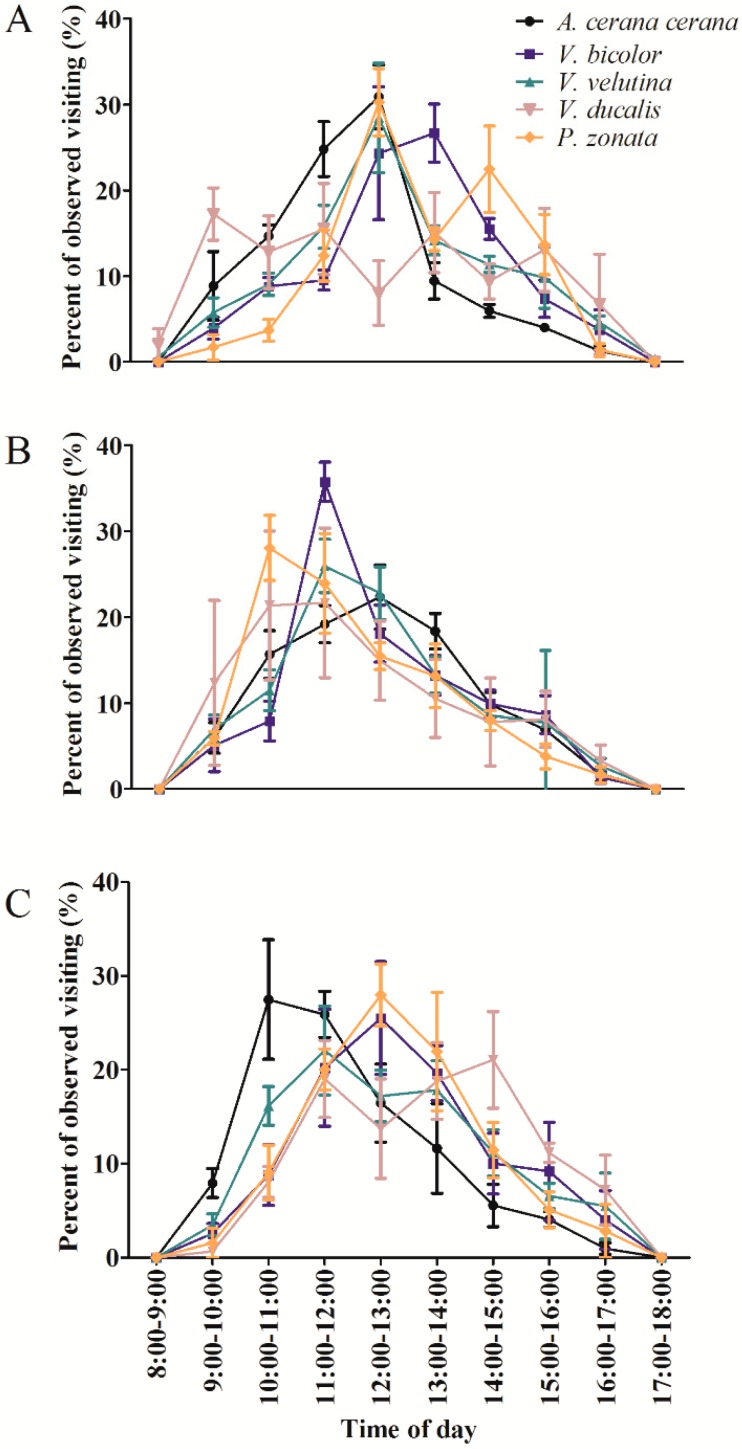
Daily activity patterns of *Apis cerana cerana*, *Vespa bicolor*, *Vespa velutina*, *Vespa ducalis*, and *Phytomia zonata* on *Camellia* osmantha (**A**), *C. vietnamensis* (**B**), and *C. oleifera* (**C**) flowers in South China.

For *C. osmantha*, the peak visiting times of *A. cerana cerana*, *V. bicolor*, *V. velutina*, and *P. zonata* were from 12:00 to 14:00, which differed from those of *V. ducalis* (Figure 3A). The five primary visiting insect species mainly visited *C. vietnamensis* from 10:00 to 12:00 (Figure 3B). The peak times at which *A. cerana cerana*, *V. velutina*, *V. bicolor*, and *P. zonata* visited *C. oleifera* occurred between 10:00 and 13:00 (Figure 3C). Among the five main visiting insects, only *V. ducalis* showed a distinct multipeak.

### 3.2. Floral Traits of C. osmantha, C. vietnamensis, and C. oleifera

The diameter of the corolla and height and diameter of the stamens of *C. vietnamensis* were significantly greater than those of *C. osmantha* and *C. oleifera* (diameter of the corolla, F = 109.875, df = 2,89, *p* = 0.000; height of the stamens, F = 44.005, df = 2,89, *p* = 0.000; diameter of the stamens, F = 62.113, df = 2,89, *p* = 0.000). The height of the pistils of both *C. vietnamensis* and *C. oleifera* were significantly higher than that of *C. osmantha* (F = 48.465, df = 2,89, *p* = 0.000) (Table 4).

### 3.3. Nectar Volumes and Sugar Concentrations in C. osmantha, C. vietnamensis, and C. oleifera Flowers

**Figure 4:**
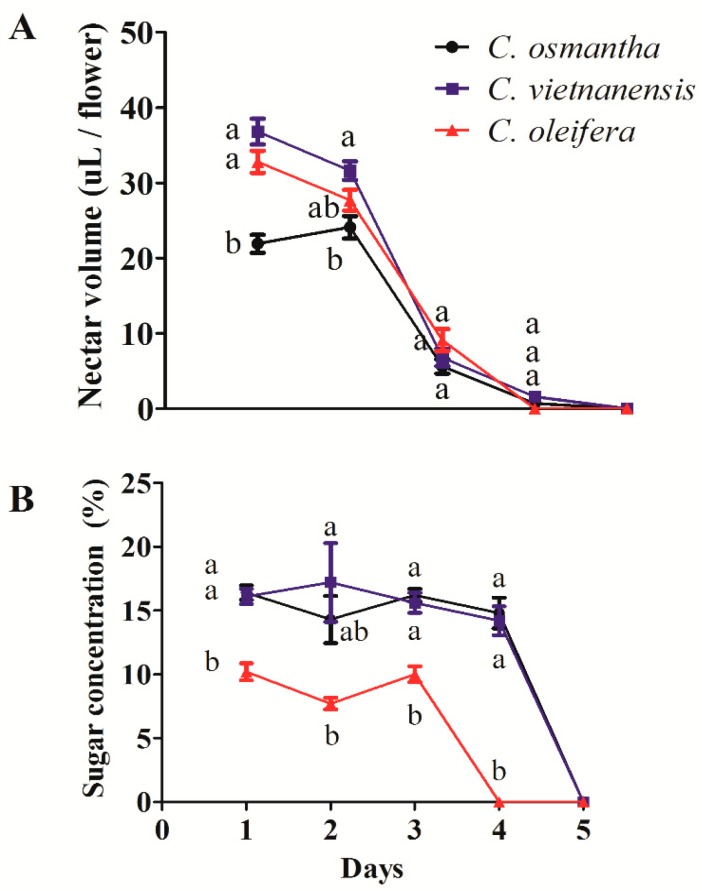
Nectar volumes (**A**) and sugar concentrations (**B**) of *C. osmantha*, *C. vietnamensis*, and *C. oleifera*. Values (mean ± S.E.) followed by different letters on the same day are significantly different based on Tukey’s HSD test at *p* < 0.05.

Nectar volumes in *C. osmantha*, *C. vietnamensis*, and *C. oleifera* flowers reached maximum values on the 1st day around florescence, and those of *C. vietnamensis* and *C. oleifera* were significantly higher than that of *C. osmantha* (F = 27.006, df = 2,8, *p* = 0.001). Additionally, nectar volumes of the three *Camellia* species gradually decreased towards zero on the 5th day (2nd, F = 7.592, df = 2,8, *p* = 0.023; 3rd, F = 2.171, df = 2,8, *p* = 0.195; 4th, F = 3.894, df = 2,8, *p* = 0.082) (Figure 4A).

The sugar concentration of *C. oleifera* was significantly lower than those of *C. osmantha* and *C. vietnamensis* on the 1st day around florescence (F = 33.631, df = 28, *p* = 0.001) even though *C. oleifera* had a higher volume. Changes in sugar concentrations were similar to the changes in nectar volume (2nd, F = 5.416, df = 2,8, *p* = 0.045; 3rd, F = 27.821, df = 2,8, *p* = 0.001; 4th, F = 76.749, df = 2,8, *p* = 0.000) (Figure 4B).

### 3.4. Effect of Insect Pollination on the Fruit Production Rates of C. osmantha, C. vietnamensis, and C. oleifera

**Figure 5:**
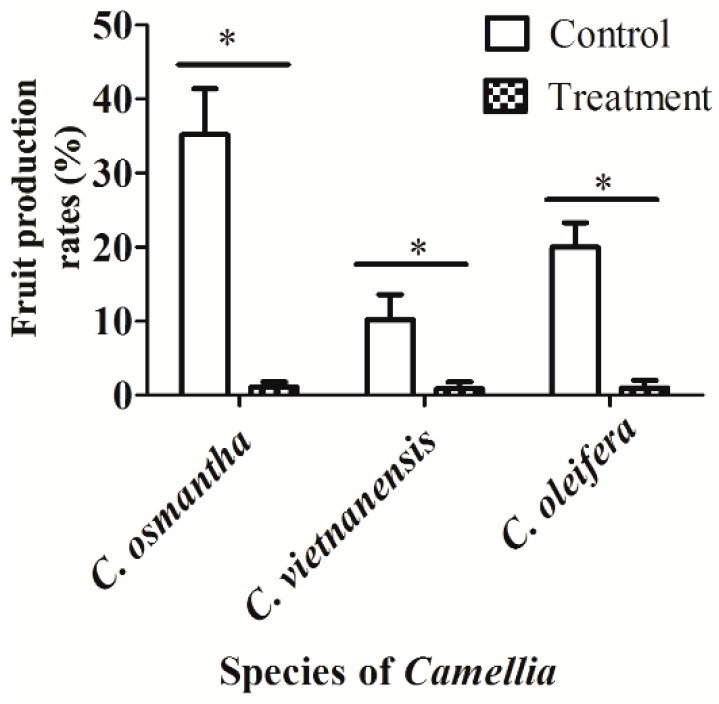
Effect of insect pollination on the fruit production rates of *Camellia osmantha*, *C. vietnamensis*, and *C. oleifera*. Asterisks indicate a significant difference between the two groups based on paired samples *t*-test at *p* < 0.05.

The fruit production rates of *C. osmantha* trees with branches that were enclosed in a net cage to prevent pollination by insects were significantly lower than those of the control (*t* = −5.732, df = 4, *p* = 0.005). There same results were observed for *C. vietnamensis* (*t* = −2.561, df = 4, *p* = 0.043) and *C. oleifera* (*t* = −5.012, df = 4, *p* = 0.007) (Figure 5). Furthermore, fruit production rates of *C. oleifera* seems to fall in-between the other two species, if pollinators are present.

## 4. Discussion

For *C. oleifera*, a total of 54 species of visiting insects, including 25 hymenopteran species, were recorded in Guangxi, South China [19,20]. Surprisingly little is known about the pollinator assemblages of *C. osmantha* and *C. vietnamensis*, although this information is crucial for ecological investigations of reproductive traits. In this study, a total of 24 species of visiting insects were identified in the field investigations, and the dominant insects visiting *C. osmantha*, *C. vietnamensis*, and *C. oleifera* included *A. cerana cerana*, *V. bicolor*, *V. velutina*, *V. ducalis*, and *P. zonata*. Although the visiting insects in this study were less abundant than those in previous reports, which may be relative to the geographical site and investigated method, the dominant species of visiting insects were consistent [19,20].

Three out of the five dominant common flower visitors to *C. osmantha*, *C. vietnamensis*, and *C. oleifera*, including the most dominant one, did not show a preference for one species. In contrast, *V. bicolor* and *V. ducalis* had an obvious preference for *C. oleifera*, although the sugar concentration of *C. oleifera* nectar was significantly lower than that of *C. osmantha* and *C. vietnamensis* nectar during flowering. Although nectar volumes in *C. oleifera* were higher than in *C. osmantha*, they did not differ from *C. vietnamensis* (Figure 4). Therefore, nectar production, which is the main source of energy for visiting insects, does not appear to be the only driver of flower preference.

Similarly, *C. vietnamensis* produces larger flowers than the other Camellia species (Table 4), but was not visited more often by any flower visitor species. Usually, larger displays of flowers/inflorescences release more floral scents and are more visible than smaller displays, attracting more visiting insects [29]. This may be explained by the theory that floral and inflorescence traits evolved under a trade-off between visual and olfactory cues [30]. In fact, odor is an important cue for visiting insects, which is often influenced by a wide variety of volatile floral scent molecules [31]. These specific floral volatile organic compound mixtures attract specialist pollinators that have evolved an innate preference for them [32]. Whether the floral scent of *C. oleifera* consists of species-specific volatile organic compounds that attract visiting insects needs further study.

Interestingly, the only dominant flower visitor species showing a clear pattern of flower constancy within a Camellia tree did not discriminate among subspecies (Table 3). In fact, flower constancy within trees was rare among dominant flower visitors, with three out of five species either visiting a single flower and leaving the tree afterwards or visiting a row of flowers consecutively. We considered that flower visitors leave an individual plant if resources are not sufficient in the flower [33] or the perception of scent marks deposited by previous visitors [34].

Many visiting insects show specific daily activity patterns [35]. In the current study, the daily activity of the five visiting insects occurred during the photophase, with clear peak visiting times from 10:00 to 14:00 (Figure 3). By timing anthesis and/or the presentation of floral rewards to match the activity peaks of their most efficient pollinators, and thus to ensuring the best pollination service, plants might enhance their reproductive output via increased pollen transfer [28]. Therefore, the daily patterns of these visitor activities may be related to the pattern of floral resource production in *C. osmantha*, *C. vietnamensis*, and *C. oleifera*. A previous study showed that the average volume of nectar in flowers exposed to pollinators varied somewhat throughout the day, reaching a maximum around midday [28]. The daily change in nectar may explain the dynamics of the visiting insects’ activity in this study. In fact, daily patterns of floral resource presentation, particularly nectar presentation, have been investigated in detail for many species, and these patterns have often been examined in relation to daily variations in pollinator activity [28,36,37]. We believe that flower visitors followed maximum nectar production either by experience or by spending more time on rewarding flowers, thereby increasing the detection probability.

*Camellia* spp. are self-incompatible and show high fruit production rates after cross-pollination. There is also increasing evidence that cross-pollination by insects increases the fruit production rates of these species [21]. The data indicated that these dominant visiting insects play a very important role in the yield of *C. osmantha*, *C. vietnamensis*, and *C. oleifera* (Figure 5). In fact, flower thinning had no significant effects on fruit production rates, e.g., in *C. osmantha* (Table S1). Thus, our results support the view that cross-pollination by insects increases fruit production rates.

## 5. Conclusions

In summary, our study examined the five species of insects, namely, *A. cerana cerana*, *V. bicolor*, *V. velutina*, *V. ducalis*, and *P. zonata*, visiting *C. osmantha*, *C. vietnamensis*, and *C. oleifera* in Guangxi, South China. The peak visiting times of these insects occurred from 10:00 to 14:00. Fruit production rates significantly increased after cross-pollination by these insects.

## Figures and Tables

**Table 1:** Species of insects visiting *Camellia osmantha*, *C. vietnamensis,* and *C. oleifera*.

Order	Family	Species	Observed Number		
			*C. osmantha*	*C. vietnamensis*	*C. oleifera*
Hymenoptera	Apidae	*Apis cerana cerana* Fabricius	412	416	380
	Halictidae	*Lasioglossum* sp.	0	0	1
	Vespidae	*Vespa bicolor* Fabricius	244	347	408
		*V. velutina* Lepeletier	350	457	430
		*V. ducalis* Smith	109	162	230
		*V. mocsaryana* du Buysson	1	6	9
		*V. velutina nigrithorax* du Buysson	7	6	22
		*V. affinis* L.	15	35	29
	Ichneumonidae	*Xanthopimpla pedator* Fabricius	0	0	1
	Formicidae	*Dolichoderus sibiricus* Emery	1	0	0
Diptera	Syrphidae	*Phytomia zonata* Fabricius	165	177	158
		*Eristalis cerealis* Fabricius	12	14	10
		*Episyrphus balteatus* de Geer	9	12	12
		*Syrphus ribesii* L.	5	8	12
		*Eristalinus arvorum* Fabricius	4	6	7
	Calliphoridae	*Calliphora vicina* Robineau-Desvoidy	3	0	5
		*Chrysomya megacephala* Fabricius	7	16	15
		*Graphomya maculate* Scopoli	3	5	8
		*Neomyia timorensis* Robineau-Desvoidy	0	0	1
Lepidoptera	Satyridae	*Melanitis leda* L.	1	0	0
		*Junonia almana* L.	0	1	0
	Pieridae	*Pieris rapae* L.	1	0	0
	Arctiidae	*Syntomoides imaon* Cramer	2	1	0
Coleoptera	Coccinellidae	*Propylaea japonica* Thunberg	2	3	2

**Table 2:** Number of insects visiting *Camellia osmantha*, *C. vietnamensis*, and *C. oleifera* flowers.

Species	Number of Insects Visiting on Flowers (Visits/Day) ^ξ^		
	*C. osmantha*	*C. vietnamensis*	*C. oleifera*
*A. cerana cerana*	51.8 ± 10.5 a	100.5 ± 35.4 a	95.0 ± 26.6 a
*V. bicolor*	43.3 ± 9.3 b	36.5 ± 13.4 b	102.0 ± 23.9 a
*V. velutina*	117.3 ± 19.1 a	80.3 ± 20.0 a	107.5 ± 40.8 a
*V. ducalis*	15.3 ± 3.6 b	14.0 ± 4.4 b	57.5 ± 15.1 a
*P. zonata*	26.8 ± 7.5 a	59.0 ± 20.1 a	37.0 ± 7.3 a

^ξ^ Number of visits by each dominant pollinating insect to *C. osmantha*, *C. vietnamensis*, and *C. oleifera* flowers on one *Camellia* tree each day. Values (mean ± S.E.) followed by different letters in the same row are significantly different based on Tukey’s HSD test at *p* < 0.05.

**Table 3:** Percentage of insects visiting the number of *Camellia* flowers.

Species	Percentage of Insects Visiting on Flowers (%) ^ξ^		
	1 Flower	2 Flowers	≥3 Flowers
*A. cerana cerana*	17.2 ± 5.4 b (154)	11.2 ± 2.2 b (103)	71.6 ± 6.8 a (732)
*V. bicolor*	27.5 ± 7.6 ab (290)	12.5 ± 4.5 b (88)	60.0 ± 7.1 a (474)
*V. velutina*	28.2 ± 10.4 ab (436)	11.0 ± 2.7 b (140)	60.8 ± 7.9 a (817)
*V. ducalis*	41.4 ± 3.4 a (85)	13.6 ± 2.4 b (28)	45.0 ± 2.9 a (96)
*P. zonata*	42.5 ± 9.7 a (198)	15.6 ± 1.5 a (80)	42.0 ± 8.6 a (215)

^ξ^ The percentage of visits by one pollinating insect to 1, 2, and ≥3 flower(s) on one Camellia tree relative to the total number of visits. Values (mean ± S.E.) followed by different letters in the same row are significantly different based on Tukey’s HSD test at *p* < 0.05. Numbers in parentheses represent sample sizes.

**Table 4:** Morphological measurements of *C. osmantha*, *C. vietnamensis*, and *C. oleifera* flowers.

Floral Traits	*C. osmantha*	*C. vietnamensis*	*C. oleifera*
Diameter of corolla (mm)	63.3 ± 1.3 c	97.8 ± 2.4 a	86.1 ± 1.1 b
Height of stamens (mm)	10.5 ± 0.3 c	15.3 ± 0.5 a	13.8 ± 0.3 b
Diameter of stamens (mm)	14.4 ± 0.6 c	25.4 ± 1.0 a	16.8 ± 0.5 b
Height of pistils (mm)	11.6 ± 0.3 b	15.3 ± 0.5 a	15.3 ± 0.2 a

Values (mean ± S.E.) followed by different letters in the same row are significantly different based on Tukey’s HSD test at *p* < 0.05.

## Supplementary Materials

The following are available online at https://www.mdpi.com/2075-4450/10/4/98/s1, Table S1: Effect of flower thinning on the fruit production rate of *C. osmantha*.
