# Supplementary material for: Diversity, Daily Activity Patterns, and Pollination Effectiveness of the Insects Visiting Camellia osmantha, C. vietnamensis, and C. oleifera in South China
Source: Insects. 2019 Apr 2;10(4):98. doi: 10.3390/insects10040098 (PMC6523973; doi:10.3390/insects10040098)
Supplement: Supplementary file 1 [file insects-10-00098-s001.pdf]

insects MDPI

Supplementary Materials

1. Methods

1.1. Effect of flower thinning on the fruit production rate of C. osmantha

Camellia osmantha branches with similar bud numbers were selected randomly in the fields (1 branch / tree). One-fourth, 1/3 and 1/2 of the total number of buds on the branches were removed before flowering (treatment groups). Branches with no bud removed served as a control group. There were five replicates for each treatment.

1.2. Statistical analysis

Statistical analysis was performed using SPSS 16.0 (SPSS Inc., Chicago, Illinois, USA). Fruit production rate of *C. osmantha* after flower thinning and control group were analyzed by one-way analysis of variance (ANOVA), followed by Tukey's honest significant difference (HSD) test for multiple comparisons. Proportional data were subjected to arcsine square root transformation prior to analysis. A level of $p < 0.05$ was accepted as statistically significant for all statistical analyses.

2. Results

2.1. Effect of flower thinning on the fruit production rate of C. osmantha

Flower thinning had no significant effect on the fruit production rate of *C. osmantha* in comparison with control ($F = 0.538$, df = 3,19, $p = 0.663$) (Table S1).

Table S1. Effect of flower thinning on the fruit production rate of *C. osmantha.*

	**Control**	**1/4**	**1/3**	**1/2**
Fruit production rate (%)	48.1 ± 9.0 a	50.3 ± 8.2a	60.9 ± 5.7 a	59.6 ± 11.4 a

Values (mean ± S.E.) followed by different letters in the same row are significantly different based on Tukey's HSD test at $p < 0.05$.
